# Drop Hammer Impact Ignition Experiment and Effect of Additives on Energy Release Characteristics of PTFE-Based Reactive Materials

**DOI:** 10.3390/polym17223029

**Published:** 2025-11-14

**Authors:** Junming Yuan, Jiaying Gu, Zhe Zhai, Jinying Wang, Peijiang Han, Jiangqi Linghu, Yang Liu

**Affiliations:** 1 School of Environment and Safety Engineering, North University of China, Taiyuan 030051, China; 17835603572@163.com (J.G.); wj1872691402@163.com (J.W.); 15835467359@163.com (P.H.); 17200631536@163.com (J.L.); 15735920620@163.com (Y.L.); 2 China Research and Development Academy of Machinery Equipment, Beijing 100089, China; zhaizhe2020@126.com

**Keywords:** PTFE-based reactive materials, aluminum alloy, drop hammer impact, energy release efficiency, ignition mechanism, explosive

## Abstract

To solve the problem of low energy release efficiency of fluoropolymer-based reactive materials, four PTFE (Polytetrafluoroethylene) -based reactive structural materials with different contents were prepared by adding traditional energetic materials (RDX, 1,3,5-Trinitrohexahydro-1,3,5-triazine) and alloy metals (aluminum magnesium, aluminum magnesium zinc). In addition, in order to reduce the high cost of the existing high-speed impact energy release testing device, the formulation optimization of PTFE-based aluminum alloy reactive material was efficiently carried out using a small-scale drop hammer impact test in this paper. The self-designed impact energy release testing device was established for the overpressure measurement of PTFE-based aluminum alloy reactive materials. The impact response processes of PTFE-based aluminum alloy reactive material were recorded with high-speed photography. The energy release characteristics were quantified using overpressure measurements. Based on the chemical reaction properties and microstructural characterization of the PTFE-based reactive materials, the ignition mechanism of aluminum alloy reactive materials was analyzed under drop hammer impact load. The results show that the quantitative characterization of the overpressure changes of reactive materials in a quasi-enclosed space before and after reaction can reflect their energy release efficiency under low-velocity impact by using the drop hammer impact energy release testing device. The order of impact response overpressure values for four PTFE-based reactive materials has been conducted. The aluminum alloy reactive material containing RDX explosive has the highest overpressure value and the highest energy release efficiency in terms of drop hammer impact response. Based on the ignition mechanism and energy release characteristics of these four PTFE-based reactive materials, it was found that the addition of alloy metal powder can reduce impact sensitivity, but when activated, it can effectively enhance the damage effect.

## 1. Introduction

Conventional combatants/munitions in service primarily utilize inert materials to damage targets through their mechanical penetration. The unique kinetic damage mechanisms and damage modes of inert metals significantly limit the power and effective damage enhancement of the combatant. In order to strike the target efficiently, the traditional fragmentation combatant, which uses kinetic energy for damage, can no longer meet the requirements. The United States first introduced the concept of the reactive damage element [1]. Reactive materials are an emerging class of high-efficiency destructive energetic structural materials, the use of which, and the preparation of reactive fragmentation of battlefield targets, can be highly effective in the implementation of destruction, with common reactive fragmentation of aluminum thermite, intermetallic compounds, metal/polymer composites, intermolecular compounds of the substable state, and hydrides [2].

Metal/fluorine polymer reactive materials are a kind of impact-induced reaction materials, which are more popular in the field of energetic fragments [3]. Open literature research shows that the metal-type reactive fragmentation combat part has obvious comprehensive destructive effects on targets [4]. Compared to traditional reactive materials such as launching charges, propellants, explosives, and pyrotechnics, metal/polymer-activated materials present more inert, significantly enhanced structural strength aspects in production, processing, storage, and transportation. The materials have a certain ability to invade and a strong loading role in the occurrence of chemical reactions.

Aluminum metal particles are often used as metal additives in reactive materials, which have high energy density and low cost and safety. However, they also have incomplete conversion of chemical energy in reactive materials. Thus, the low energy release efficiency of reactive materials is one of the problems that needs to be solved nowadays.

Ding [5] added CuO to PTFE/Al reactive materials and evaluated the impact energy release capacity of such reactive materials through drop hammer experimental studies with ternary reactive materials as the main object of study. The experimental results showed that the increase in aluminum thermite content could help to promote the reaction of reactive materials. But the overall energy release efficiency was low (about 6~20%).

He [6] successfully prepared core-shell structured Al/PTFE composites through mechanical activation and sintering techniques to improve their combustion performance and energy release rate. The Al/PTFE composites showed significantly enhanced combustion performance. Pmax and flame propagation speed increased by 119% and 349%. Notably, Pmax/Δt reached 11.30 times, indicating a significant enhancement in reaction activity. Xiong [7] prepared Al/PTFE composite fuels with different PTFE contents (3 wt%, 8 wt%, 15 wt%). The study found that the PTFE coating can effectively promote energy release from aluminum fuel. Among them, the Al/PTFE_8% fuel exhibited more stable thermal performance and a higher weight gain rate. The ignition delay of this sample was reduced by 41.9%. The combustion temperature increased by 17.1%.

Currently, there are two main strategies to improve the energy release of reactive materials: one involves reactive material preparation processes, such as molding and sintering processes [8], and mechanical force activation methods [9]. And the second strategy involves adjusting the formulation of the reactive materials, such as increasing metal oxides (Bi_2_O_3_, Fe_2_O_3_), and using nano-metallic aluminum to reduce the particle area [9].

Jiang [10] prepared PTFE/Al/Bi_2_O_3_ reaction material and analyzed the impact of particle content on it. The results showed that the duration and sensitivity of the shock-induced chemical reaction first increased and then decreased with increasing Bi_2_O_3_ content. The highest shock sensitivity and the longest reaction time were observed when the Bi_2_O_3_ content was 9%. Crack tip heating was responsible for the ignition of the reactive material under long pulses of low-velocity shocks.

Huang [11] studied the mechanical properties and impact energy release characteristics of a ternary reactive material (PTFE/Al/Fe_2_O_3_). In a drop hammer impact test, an explosion and combustion occurred. And the detection of the reaction products indicated that an aluminothermic reaction occurred. This shows that the addition of oxides is a feasible approach, but it cannot indicate whether energy release efficiency is improved. Seropyan [12] and others proposed that certain oxide matrices can also be added as additives to reactive materials. At the same time, ammonium perchlorate (AP) and potassium perchlorate (KP) can also be added to reactive materials.

Introducing traditional energetic structural materials and high-calorific-value alloy materials into reactive materials is a simple and effective method. Cao [13] investigated the energetic performance of HMX (Octahydro-1,3,5,7-tetranitro-1,3,5,7-tetrazocine) -based aluminum-containing explosives with the addition of PTFE oxidizers. Compared with the aluminum-containing explosives without PTFE, the addition of PTFE to the aluminum-containing explosives significantly increased the heat of explosion in terms of the calorimetric data.

Wang [14] significantly increased the energy output, pressurization rate, and burning rate (8863 J/g, 340 MPa/ms, and 1626 m/s) by adding 9 wt% ammonium perchlorate to the PTFE/Al reaction material, which was much higher than that of pure PTFE/Al (2019 J/g, 29.3 MPa/ms, and 260 m/s, respectively). Huang [15] prepared aluminum-based microspheres containing CL-20 (Hexanitrohexaazaisowurtzitane) by electrospray deposition. Their surface morphology could be adjusted using different polymers as binders. Thermal behavior results showed that CL-20 had no significant effect on the reactivity of the aluminum nanoparticles. The fluoropolymer binder (F_2314_) promoted the oxidation of nano-aluminum and the presence of CL-20 could significantly improve the reactivity of nano-aluminum in combustion behavior tests.

Song [16] improved the sensitivity and energy release characteristics of Al/PTFE composites by introducing the strong oxidizer KMnO_4_ and the reducer TiH_2_. When the potassium permanganate content reached 40 wt%, the Al/PTFE/TiH_2_/KMnO_4_ reactive material exhibited the highest sensitivity and compressive strength, with a dynamic compressive strength of 129.12 MPa. This represents an increase of 40.62% compared with unsintered Al/PTFE composites and an increase of 406.2% under quasistatic compression conditions. Ao [17] showed that by introducing five different aluminum-based alloys, Al/Mg, Al/Ni, Al/Si, Al/B, and Al/Zn, into the reactive materials, the test results based on the thermogravimetric/differential scanning calorimetry (DSC) method showed that all five alloys could increase the initial oxidation temperature.

Zhao [18] compared the reactive properties of two reactive materials, Ni/Al/Al_12_Mg_17_ and Ni/Al. The results showed that the rapid oxidation of liquid magnesium plays a key role in the thermal explosion reaction in air. During the self-propagating combustion process, the Al_12_Mg_17_ additive acts as an ‘ignition point.’ The combustion of Mg ignites the Ni/Al intermetallic reaction. The continuous power provided by Mg combustion promotes sustained reactions within the Ni/Al matrix. The combustion wave velocity of the Ni/Al/Al_12_Mg_17_ material nearly doubles, increasing from 60–100 to 150–200 mm·s^−1^. This indicates that the Al_12_Mg_17_ additive is suitable for enhancing the performance of Ni/Al-based reactive materials. Therefore, combining PTFE/Al reactive materials with conventional reactive materials is a new approach to improving energy release efficiency.

Ames [19] and Kurdyumov [20] conducted an in-depth study on the impact initiation mechanism and energy release behavior of sintered reactive materials through Taylor rod collision experiments. When the impact velocity reaches 163 m/s, the reactive material under the action of the applied load occurs local ignition and combustion. The results show that the energy release ignition of the reactive material first appeared in the strong shear deformation zone. Thus, it strongly confirms that the main mechanism of energy release of reactive material is the strong shear effect.

Cai [21] and Sharma [22] et al. tested the dynamic failure behavior of molded Al/W/PTFE ternary fluoropolymer-based reactive materials at high strain rates by means of drop hammer experiments and scanning electron microscopy (SEM). The test results show that the reactive material matrix PTFE is more prone to plastic deformation and cracks are more likely to occur at the demarcation of matrix PTFE and W powder. Local shear and thermal softening caused by plastic deformation of PTFE leads to crack extension and ultimately results in macroscopic damage to the reactive material. Yang [23] studied experimentally and simulatively the dynamic compression response under loading. The results showed that the interfacial bonding interactions were relatively weak and that enhancing the interfacial strength would increase the strength of Al/PTFE composites. Further studies showed that the strength of the interfacial layer plays a key role in plastic strain localization and hotspot generation, which causes ignition of the composite.

Liu [24] and colleagues prepared composites of different particle sizes to investigate the effect of alumina thickness on the reactivity of Al/PTFE composites. The study shows that halving the thickness of the alumina shell can increase the peak diffusion rate of oxidizer atoms by 1.6 times. Geng [25] and colleagues designed Al/PTFE (26.5/73.5 wt%) samples with three inclination angles of 0°, 30°, and 45° through SHPB tests and numerical simulations based on the dynamic compression–shear ignition mechanism. The study indicates that as the angle increases, the degree of damage worsens, and the ignition threshold decreases from 0.424 J/(g·μs) to 0.377 J/(g·μs). The strain rate corresponding to ignition decreases from 11,578 s^−1^ to 11,366 s^−1^.

Qiang [26] used a self-developed high-and low-temperature testing system, combined with a universal material testing machine and SHPB experimental system, to study the mechanical properties of PTFE/Al composites under high-and low-temperature conditions. The study found that PTFE/Al composites exhibit a delayed detonation characteristic at low impact speeds, and the delay time decreases as the impact speed increases. Zhou [27] introduced the inert substitute PTFE/LiF to differentiate the contributions of kinetic energy and chemical energy of PTFE/Al reactive materials during impact. The dynamic crushing test results of PTFE/Al and PTFE/LiF samples further confirmed the potential of PTFE/LiF as an inert substitute for PTFE/Al.

In summary, reactive materials have been prepared from the preparation process, adjusting the reactive material formula and theoretical analysis of its ignition mechanism and other aspects of a more in-depth study and laying the theoretical and experimental foundation for this research in this paper. This paper is based on a low-cost small-scale drop hammer test pressure testing system. By adding Al/Mg alloy powder, low-melting-point Zn, and RDX explosive particles to the traditional PTFE/Al reactive material formulation, the reactivity and energy release characteristics of the reactive material are enhanced. In addition, based on experimental test results, a theoretical analysis is conducted on the effects of additives such as aluminum alloy powder and reactive materials on the dynamic response behavior and impact energy release characteristics of PTFE-based reactive materials. The study also explains the impact-induced energy release process and reveals the ignition mechanism. The research results can promote the advancement of highly efficient destruction technology and protection technology and provide theoretical basis for the stability of reactive materials in the production, storage, transport, and other aspects.

## 2. Materials and Methods

### 2.1. Formulation of PTFE-Based Reactive Material

Al powder in Al/PTFE reactive materials is widely used in energetic material formulations due to its inherent reactive and combustion properties. The main research approach currently is to regulate the sensitivity and energy release characteristics of PTFE/Al reactive materials by physically coating, chemically modifying, or doping additives to their original components. Since aluminum is highly prone to oxidation, its surface forms a dense aluminum oxide shell. This leads to a decrease in the energy release efficiency of Al/PTFE reactive materials. In this study, additives such as aluminum alloy metal powders and explosive particles were doped into the PTFE-based reactive material formulations. The aim is to alter their surface properties, microstructure, composition, dispersion state, and interfacial bonding characteristics, thereby enhancing the reactivity of the novel aluminum alloy-based reactive materials and improving their energy release efficiency. Al_2_O_3_ on the metal aluminum surface will hinder the ignition and reaction of the reactive metal aluminum inside, greatly limiting the output energy of PTFE/Al reactive materials. How to reduce the oxide layer on the reactive material energy release is a major problem that needs to be solved. The use of PTFE/Al based on the release of better aluminum alloy metal powder and the low melting point of the metal can effectively enhance the energy output [28]. The combustion heat of pure Al powder is about 31 kJ/g. The combustion heat of Al/Mg alloy powder is about 30.5 kJ/g. The combustion heats of the two are similar. The melting point of Al/Mg alloy powder is about 463 °C. This temperature is significantly lower than the melting points of pure aluminum (660 °C) and pure magnesium (651 °C). This is a typical characteristic of low-eutectic alloys. The addition of Al/Mg alloy powder effectively lowers the material’s impact ignition threshold and ensures stable propagation of the reaction. This helps to complete the combustion reaction.

Aluminum alloy reactive material release energy is affected by parameters such as alloy type and dosage. The low-melting-point metal powders such as Zn can first generate metal vapor in a high-temperature impact environment, thereby creating a localized high-temperature and high-pressure condition [29]. This helps remove the inert protective layer on the surface of aluminum particles and increase their reactivity to enhance the intensity of the reaction. The introduction of organic reactive materials increases reactive gas yields. It has been shown that the introduction of nitrocellulose (NC) into substable intermolecular complexes can significantly improve gas production and increase pressure output [30]. In this experiment, the explosive RDX was chosen as an additive, similar to nitrocellulose, with the consideration of increasing the system’s reactive pressure output. In addition, RDX particles exhibit the characteristic of undergoing combustion and detonation under high-speed impact. Therefore, in PTFE-based aluminum alloy reactive materials, RDX is more important as an energetic component, forming hotspots in the early reaction stage to enhance the system’s reactivity. To explore the effect of different additives on the energetic reactive materials, the materials for this test are shown in Table 1. The raw materials used in this experiment include Al, D_50_ = 5 μm; PTFE, D_50_ = 10 μm; Al/Mg (10/90), D_50_ = 30 μm; Al/Mg/Zn (10/80/10), D_50_ = 30 μm; RDX, D_50_ = 10 μm.

### 2.2. Preparation of PTFE-Based Reactive Material

**Figure 1:**
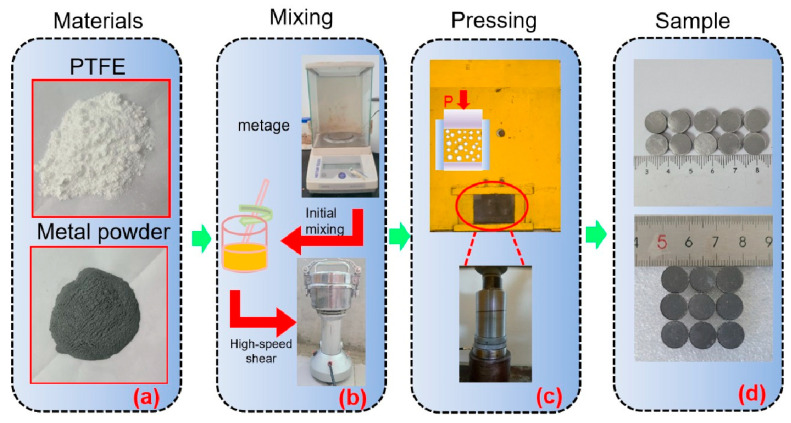
The sample preparation process of the PTFE-based reactive material: (**a**) raw materials; (**b**) mixing process; (**c**) suppression process; (**d**) formed sample.

The optimum reaction mass ratio of PTFE to Al was 73.5:26.5. The test was firstly weighed accurately with an electronic balance to place PTFE powder and Al powder in a beaker and stir the initial mixing for 30 min. Then the mixture was put into a high-speed shear to mix for 10 min and left to stand under natural conditions for 24 h. The test was carried out with a high-speed shear with the help of an electronic balance. The molding pressure was 40 MPa, the holding time was 30 min under the conditions, and it was kept for 1 h placed in a mold with dimensions of Φ10 mm × 10 mm when cold-press forming (25 °C), as shown in Figure 1.

### 2.3. TGA-DSC Combined Testing Method of PTFE-Based Reactive Material

Thermogravimetric analysis–differential scanning calorimetry (TGA-DSC) was utilized to analyze the thermal properties of four different formulations of PTFE-based aluminum alloy reactive materials in this paper. By analyzing the test results, the thermal effects of different formulations can be compared, and their thermal stability can be evaluated. Studying the thermal properties of PTFE-based aluminum alloy reactive materials is of great significance for subsequent research and application of reactive materials.

A simultaneous thermal analyzer (TGA-DSC) can provide TG and DSC signals simultaneously. A single measurement can obtain both mass change and thermal effect information. The TG signals are used to measure reactive material mass changes, composition analysis, thermal stability, decomposition behavior, and more. DSC signals are used to measure material melting/crystallization, solid-phase transitions, glass transitions, crystallinity, etc. TGA-DSC analysis can determine the physical or chemical processes corresponding to a thermal effect based on whether the thermal effect is associated with mass change. In this study, a Swiss Mettler TGA-DSC 1 simultaneous thermal analyzer was used. Its calorimetric accuracy is ±1%, temperature accuracy is ±0.3 °C, balance sensitivity is 0.1 µg, heating rate range is 0.1–100 °C/min, and measurement temperature range is 0–1500 °C. According to GJB 772B-2022 “Explosive Test Methods,” [31], the test temperature was increased from 30 °C to 800 °C at a heating rate of 10 °C/min. The test was conducted in a nitrogen atmosphere with a sample mass controlled to around 5 mg.

### 2.4. Drop Hammer Impact Ignition Experiment Test of PTFE-Based Reactive Material

**Figure 2:**
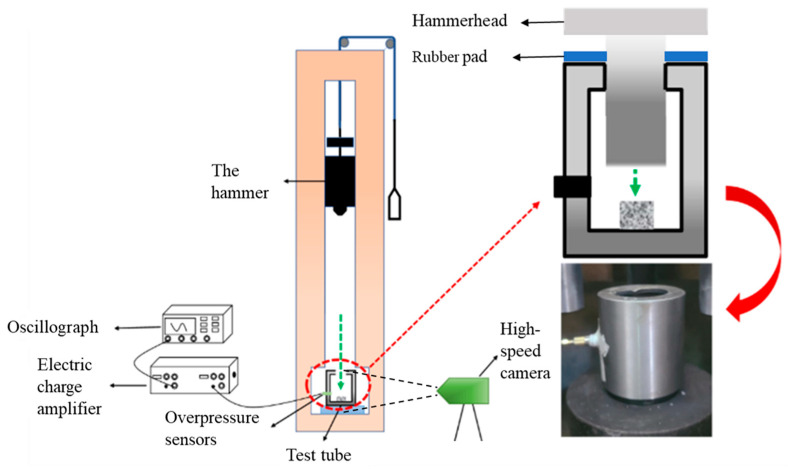
Composition diagram of a self-designed impact energy release testing device.

Energetic structural materials undergo chemical reactions and release a large amount of energy under impact. Its energy release characteristics are significantly different from those of slow thermal analysis experiments. Ames [32] pioneered the use of quasi-closed containers to quantitatively characterize the energy release properties of reactive materials. This paper references the principle of the quasi-enclosed apparatus of the Ames test system for scaled-down design experiments and designs an energy release test device. Different formulations of fluoropolymer based reactive materials were carried out using a self-designed impact energy release testing device to quantitatively characterize the ability of the reactive materials to release energy on impact and the experimental field setup, which is shown schematically in Figure 2.

The drop hammer impact pressure testing device mainly includes a drop hammer impact instrument, a quasi-compact test system, an oscilloscope, a charge amplifier, an overpressure transducer, a high-speed photographic camera, and a quasi-closed container with dimensions of Φ 100 mm × 45 mm. The parameters of the sensor used in the drop hammer impact pressure testing device are shown in Table 2.

The formula for calculating pressure is as follows:

(1) $$∆P=\frac{V}{K_{1}K_{2}}-\frac{b}{K_{2}}$$

where ∆*P* is the measured pressure, kPa; *V* is the measured output voltage, mv; *K*_1_ is the charge amplifier gain, mv/pC; *K*_2_ is the sensor sensitivity, pC/kPa; *b* is the sensor offset, pC.

A drop hammer is used to impact the reactive material to stimulate the reaction of the reactive material, and then the pressure in the vessel rises; the mass of the hammer is 10 kg. The maximum stroke of the drop hammer system is 150 cm. Therefore, the experiment uses a drop height of 100 cm to impact the reactive material tablets with the hammer. Ignoring the friction of the hammer on the sliding track, the hammer’s speed at the moment of impact after free falling from a height of 100 cm is approximately 4.43 m/s. This process is considered a low-speed impact. Given the characteristics of the device, the sample size of the reaction material used in this paper is Φ 10 mm × 3 mm. The sample itself has a small mass of 1.82 g and a density of approximately 2.3 g/cm^3^. The RDX content accounts for only five percent. At the same time, the pressure in the entire explosion chamber cannot exceed 0.1 MPa. During the overpressure testing experiment, steel plates were also used as a good protective measure to ensure safety. Therefore, the entire experiment was safe and controllable.

An overpressure transducer was placed at the same level as the reactive sample to quantitatively characterize the ability of the reactive material to release energy on impact, while a high-speed camera was used to record the ignition impact process. Prior to the experiment, the samples were placed between quasi-closed test cylinders. At the start of the experiment, the samples were held in a constant position. Subsequently, by pulling the mechanism, the drop hammer impacts with the sample, while the high-speed camera is activated and the whole impact process is completed.

## 3. Results and Discussion

### 3.1. The TGA-DSC Test Results Analysis of PTFE-Based Reactive Materials

**Figure 3:**
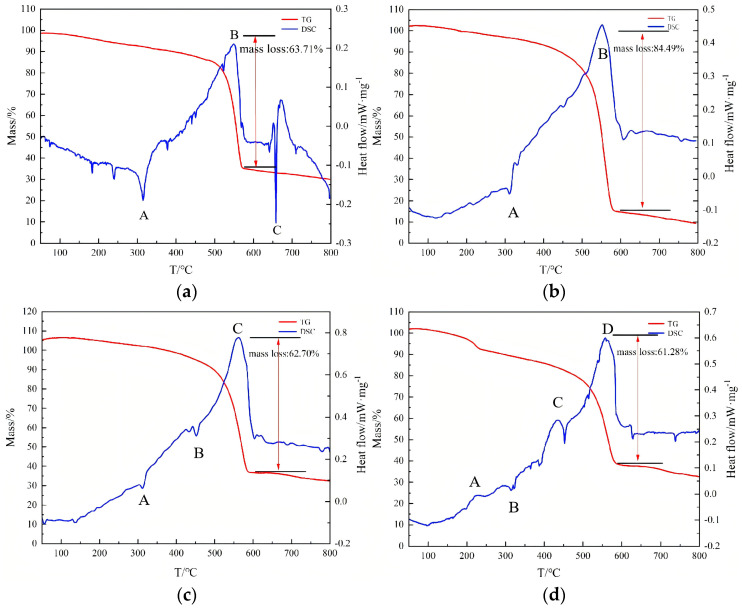
The corresponding thermal characteristic curves of PTFE-based aluminum alloy reactive materials: (**a**) sample 1#; (**b**) sample 2#; (**c**) sample 3#; (**d**) sample 4#.

A synchronous thermal analyzer was used to perform TGA-DSC tests on four formulations of reactive materials. The corresponding thermal characteristic curves of PTFE-based aluminum alloy reactive materials for the four formulations are shown in Figure 3.

Figure 3a shows the TGA-DSC curve of the Al/PTFE reactive material sample. The DSC curve (blue) exhibits an endothermic peak A between 300 and 350 °C, corresponding to the melting endothermic process of PTFE. The TG curve (red) shows a rapid decline starting at around 550 °C until 600 °C. And simultaneously, the DSC curve shows a significant exothermic peak B around 550 °C, indicating the reaction between PTFE and Al. The sample experiences a mass loss of approximately 63.71%. As the temperature continues to rise to 660 °C, the unreacted metallic Al undergoes melting endotherm, corresponding to the endothermic peak C observed in the DSC curve.

Figure 3b shows the TGA-DSC curve of the PTFE/(Al/Mg) reactive material sample. The DSC curve shows an endothermic peak A between 300 and 350 °C, corresponding to the melting endothermic process of PTFE. Around 550 °C, a strong and sharp exothermic peak B appears. At this time, a large amount of reactive fluorine produced by the decomposition of PTFE reacts violently with the molten Al/Mg, forming metal fluorides and releasing a large amount of heat. The TG curve shows a sample mass loss of approximately 84.49%.

Figure 3c shows the TGA-DSC curves of the Mg/Zn/Al/PTFE reactive material sample. Endothermic peak A corresponds to the melting of PTFE. As the temperature rises to 420 °C, reaching the melting point of metallic Zn, the corresponding endothermic peak B appears in the DSC curve. When the temperature reaches around 510 °C, PTFE begins to undergo endothermic decomposition. As the temperature continues to rise to about 562 °C, exothermic peak C appears. This is due to the vigorous exothermic reaction between Mg and PTFE, which masks the endothermic decomposition process of PTFE. The TG curve shows that the sample’s mass loss is about 62.7%.

Figure 3d shows the TGA-DSC curves of the Mg/Zn/Al/PTFE reactive material sample. An exothermic peak A occurs at approximately 200–250 °C, during which a mass loss step appears in the TG curve. This is the result of rapid decomposition of RDX producing a large amount of gas. The DSC curve exhibits an endothermic peak B between 300 and 350 °C, corresponding to the melting endothermic process of PTFE. An exothermic peak C appears at 420–460 °C due to the fluoro-thermal reaction between Zn and PTFE. There is a significantly weakened and broadened exothermic peak D around 558 °C. This reflects the fluoro-thermal reduction reaction between the residual PTFE and Al/Mg/Zn. At this point, the TG curve shows a mass loss of approximately 61.28%.

Sample 2# has the highest weight loss rate, indicating that its reaction is more complete. A sharp endothermic peak C is present in Figure 3a, which is caused by the melting of unreacted metallic Al. However, such a sharp endothermic peak cannot appear in the DSC curves of subsequent samples, indicating that samples 2#, 3#, and 4# reacted more completely than sample 1#.

### 3.2. The Impact Ignition and Energy Release Analysis of PTFE-Based Reactive Materials

**Figure 4:**
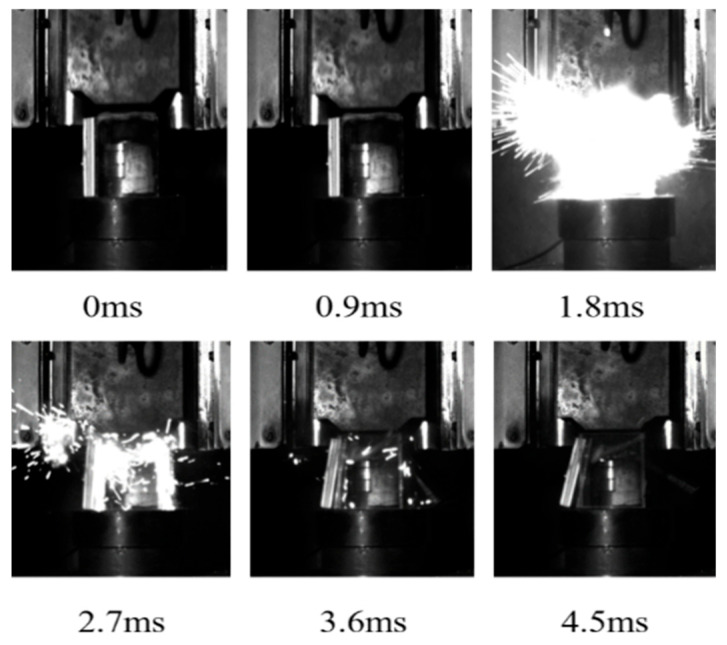
Impact energy release process of sample 1#.

**Figure 5:**
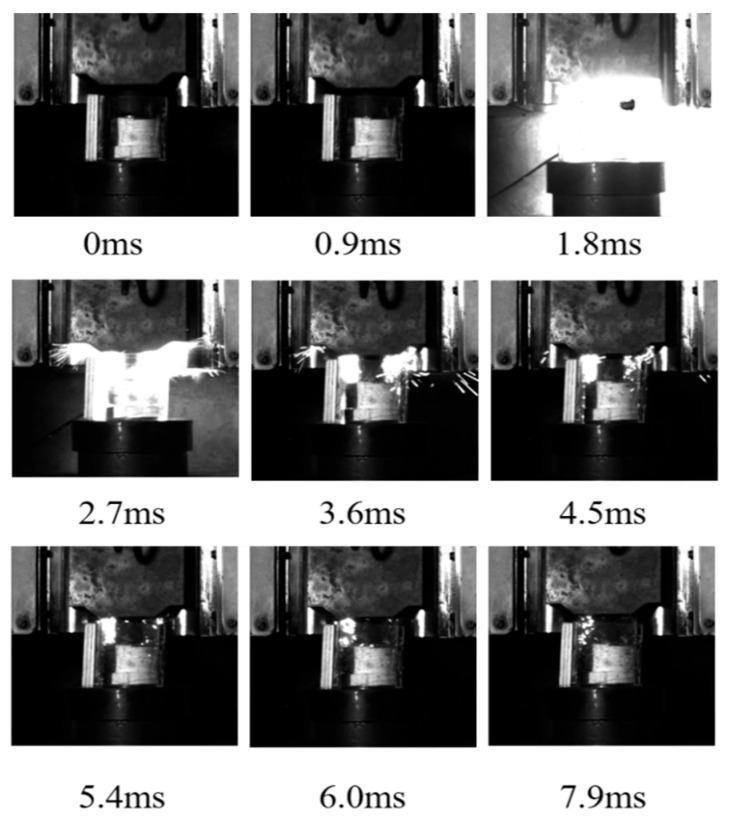
Impact energy release process of sample 2#.

**Figure 6:**
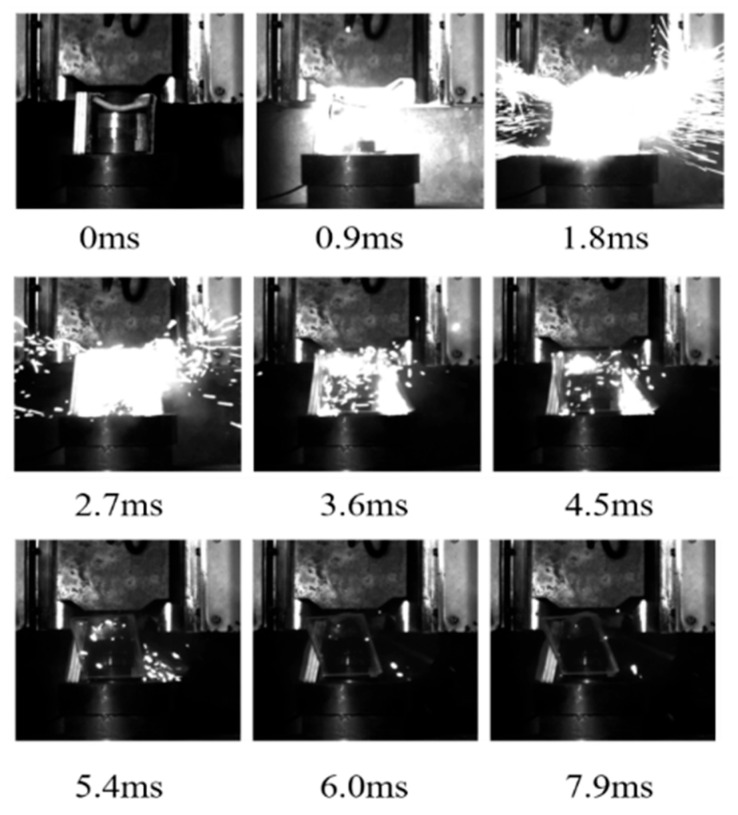
Impact energy release process of sample 3#.

The energy release process in response to the drop hammer impact of the aluminum alloy reactive material was recorded with a high-speed camera using a transparent airtight container, and typical reaction processes are shown in Figure 4, Figure 5 and Figure 6. The moment before the drop hammer touches the reactive material is recorded as the initial moment, 0 ms. The reaction inside the transparent container is the most intense and the light is the brightest after 2 ms. The impact response of the drop hammer is completed in a very short time, and a large number of fragments are burned and reacted after impact crushing. The reaction products and air mixture are ejected from the upper end of the closed container. The tremendous heat generated by the reaction increases dramatically in a short period of time. The mixture is ejected over a distance, and the light produced by the reaction also weakens as the deflagration reaction diminishes. Comparison of transparent quasi-closed test cylinders before and after the reaction showed that the reaction products adhered to the inner wall of the container. This is caused by the ejection of the fluorination products from the fluorination reaction into the wall of the container during the reaction.

As shown in Figure 4, during the impact process, the ignition of sample 1# occurred at 0–0.9 ms. At this moment, the ignition phenomenon was not obvious. There was a bright flame whose diameter is about 17 cm at 1.8 ms. The reaction was rapidly extended outward. Then, the reaction gradually weakened, and the sparks were splashed around the reaction zone. The overall reaction lasted for about 5 ms.

As shown in Figure 5, during the impact process, the sample 2# was ignited when 0~0.9 ms, but the ignition phenomenon is not obvious. At 1.8 ms, there is a bright flame whose diameter is about 9 cm. The reaction quickly expanded outward. Subsequently, the reaction gradually weakened, producing a jet flame of about 100 mm. The overall reaction lasts for about 8 ms, which is longer compared to sample 1#.

As shown in Figure 6, there is a bright flame whose diameter is about 19 cm for sample 3# at 1.8 ms. The reaction extends rapidly outwards until 3.6 ms, and then the reaction gradually weakens. The overall reaction lasted around 8 ms, which is a longer reaction duration and earlier ignition time compared to sample 1#.

**Figure 7:**
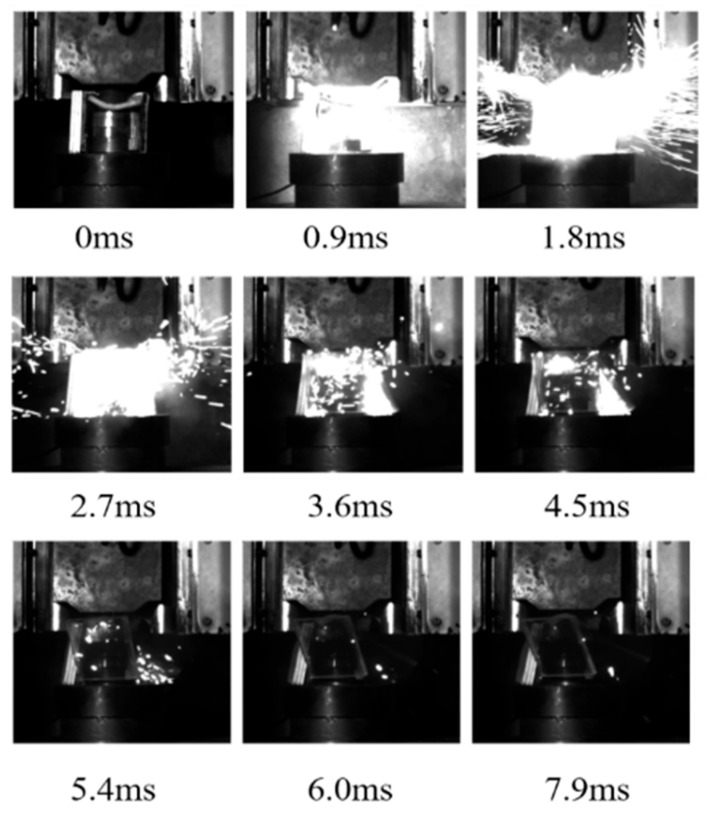
Impact energy release process of sample 4#.

As shown in Figure 7, during the impact process, at 0~0.9 ms, the ignition of sample 4# occurs and the ignition phenomenon is obvious. A bright flame with a diameter of about 22 cm is formed at 1.8 ms. The reaction rapidly expands outwards, and at the same time the brightness is obvious and lasts for 2.7 ms. Subsequently, the reaction gradually weakens. The overall reaction duration was around 8 ms, which is longer and earlier ignition time compared to sample 1#.

### 3.3. Characterization and Micromorphology of the Samples After Impact Ignition

**Figure 8:**
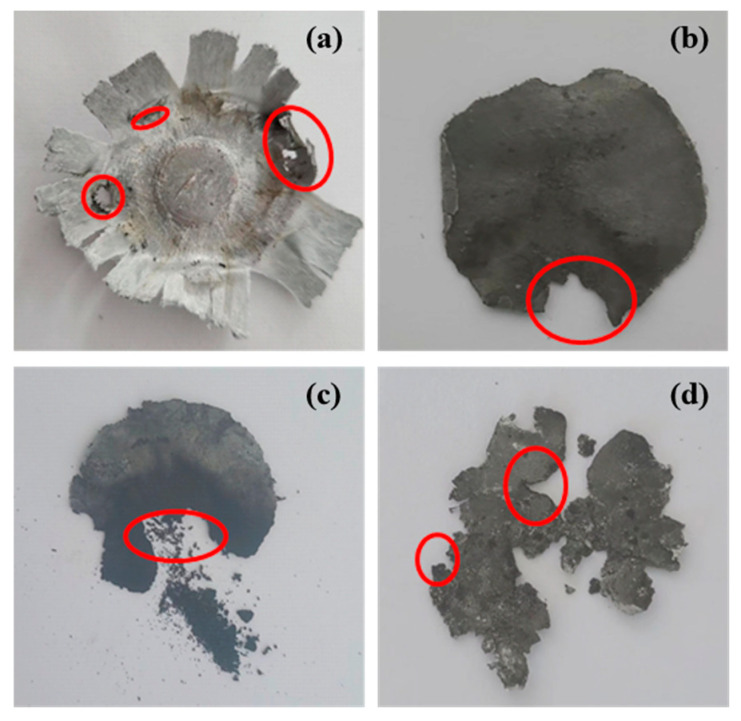
Sample diagram after impact ignition of PTFE-based reactive materials: (**a**) sample 1#; (**b**) sample 2#; (**c**) sample 3#; (**d**) sample 4#.

The PTFE-based aluminum alloy reactive material reacts on the witness plate to produce ignition and combustion marks. These phenomena indicate that the reactions are becoming increasingly intense during the generation and spread of hotspots. The combustion or explosion phenomenon of these aluminum alloy reactive reactants can propagate the reaction from one center of the tablet to another as shown in Figure 8. The red circle represents the location of the ignition and combustion marks of the reactive material.

All four types of aluminum alloy ignited under hammering. The dents in Figure 8 indicate the ignition areas located at the points of friction between the hammer head and the sample. Increased content of metal powders reduces the plasticity of the material. The material undergoes a greater degree of fracture and fragmentation, not plastic deformation [33].

**Figure 9:**
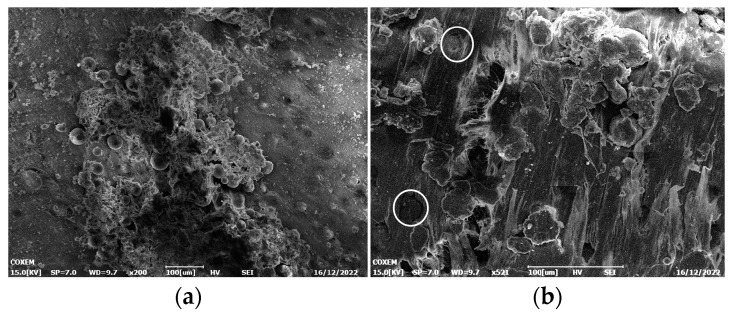

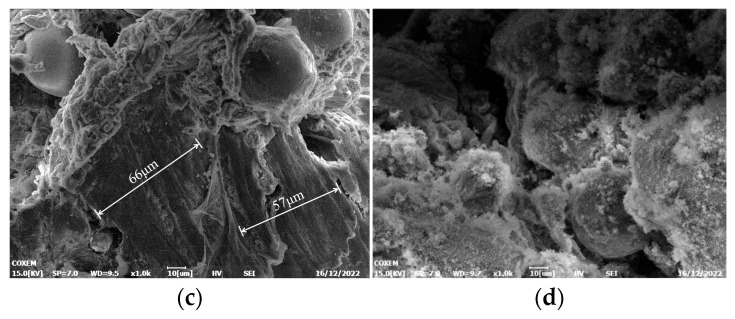
Micro-morphology after impact ignition: (**a**) sample 1#, 200× times; (**b**) sample 2#, 500× times; (**c**) sample 3#, 1.0k× times; (**d**) sample 4#, 1.0k× times.

The reactive material reacts with a high degree of fragmentation and a violent energy release process. The impact ignition phenomenon is different for different ratios of reactive materials and fillers [34]. Under the action of applied load, the metal particles undergo internal slip and friction, shear zones are rapidly generated and spread, and energy is gradually accumulated. Figure 9 shows the microscopic morphology of PTFE-based aluminum alloy reactive material. In Figure 9a, a significant strip of burning marks was produced locally on the tablet after impact ignition and reaction. Due to the occurrence of slipping, the particles experienced significant deformation, as shown in the white circle in Figure 9b. Figure 9c shows obvious shear bands with a distinct width ranging from 57 μm to 66 μm. Figure 9d shows serious tearing marks inside the sample.

During the compression process, the pores of the material are squeezed and homogenized by the external force. The reactive material is compressed. Then high pressures are generated in certain areas. The inhomogeneity of the distribution promotes the formation of hotspots. The collected ignition samples showed black reaction traces at the edges of the openings, suggesting that the reaction took place at the shear cracking point. This is consistent with the “shear-initiated reaction mechanism” of Al/PTFE-based reactive materials proposed by Lu [25,35]. The initial ignition time and combustion duration of the PTFE-based aluminum alloy reactive materials is shown in Table 3.

### 3.4. The Overpressure Test Results and Analysis of PTFE-Based Reactive Materials

The five repeated experiments have been conducted on samples of four different formulations using the overpressure test system and obtained the corresponding overpressure data. Typical overpressure test data are shown in Table 4. The results of the five repeated experiments are listed in Table 4. Due to the small sample size and the relatively small volume of the enclosed space, the sealing capability of the overpressure testing device is somewhat insufficient which may lead to lower pressure measurement results.

**Figure 10:**
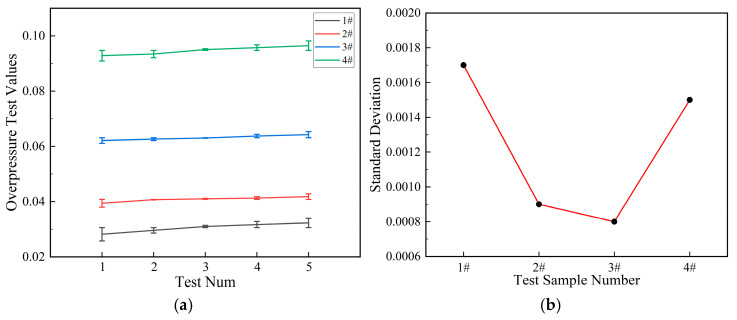
The overpressure test data chart of the four samples: (**a**) the overpressure test data with error bars; (**b**) the standard deviation curves of the overpressure peak value.

Figure 10a shows the overpressure error bars for the four samples after five repeated experiments. Figure 10b shows the standard deviation curves of the four samples.

**Figure 11:**
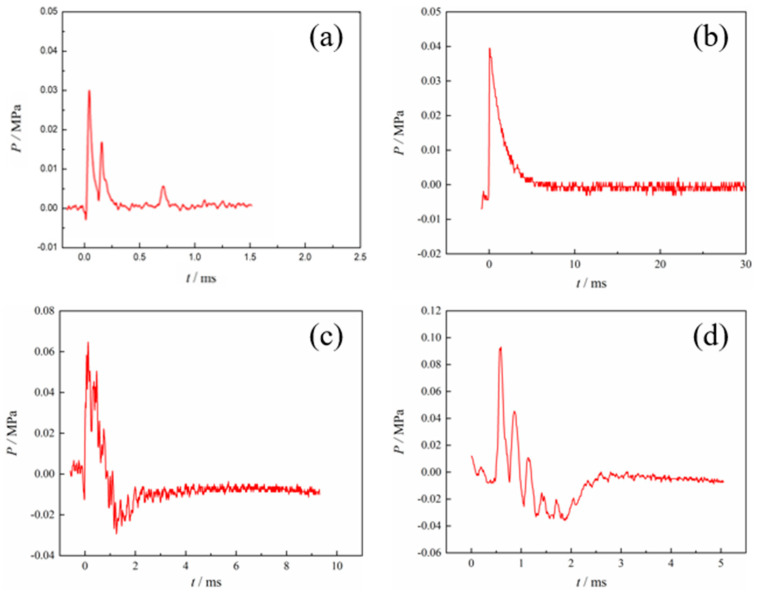
Shock wave overpressure-time curve of PTFE-based reactive materials: (**a**) sample 1#; (**b**) sample 2#; (**c**) sample 3#; (**d**) sample 4#.

As Figure 11 shows the shock wave overpressure curves of different formulations of aluminum alloy reactive materials, the relationship between the size of the overpressure peaks after the drop hammer impact initiated the reactive materials is 4# > 3# > 2# > 1#. This indicates that metal powders such as magnesium, zinc, and RDX additives promote the reaction materials. This study assumes that the reaction is complete and calculates energy efficiency using peak pressure rather than integrated pressure. The calculation results of energy release efficiency may be certain limited.

The shock waves generated during the testing of PTFE-based aluminum alloy reactive materials consist of the initial shock wave, the reaction shock wave, and the reflected shock wave. The initial reaction process of PTFE-based aluminum alloy reactive materials is similar to an explosion, as can be seen from Figure 11. The curve has a portion of negative pressure before rising, and it is accompanied by curve oscillations. This is mainly caused by the splashing of the reactive material sample. Under the impact of a falling hammer, the reactive material is activated and then a detonation-like phenomenon occurs. A large amount of energy is released in a short period of time, forming a shock wave in an enclosed space, ultimately causing the pressure inside the enclosed space to rise. As the reaction proceeds, the metal powder in the sample undergoes the “afterburning effect”, and the shock wave is reflected many times in the confined space and the overpressure curve forms a smaller overpressure peak and finally forms the curve shown in Figure 11.

The reaction of reactive substances in a closed chamber causes pressure changes, and the reactive substances can be reflected by the pressure ratio [36]. Therefore, in order to quantitatively describe the energy release efficiency, the energy release efficiency is defined as follows:

(2) $$\eta =\frac{∆P}{∆P^{*}}$$

where *η* represents the energy release efficiency, ∆*P* represents the experimental test pressure data, and ∆P* represents the theoretical pressure change.

The reaction process of reacting substances in the cavity can be regarded as an adiabatic reaction process. Combined with the ideal gas equation of state, the following relation can be obtained [2]:

(3) $$∆P^{*}=\frac{\gamma -1}{V}∆E$$

where *γ* represents the specific heat capacity ratio of the air in the cavity, which is taken as 1.40; *V* is the effective volume of the cavity; and ∆*E* is the total energy of the reactive substances deposited in the cavity. This experiment calculates the energy released based on complete reaction. According to the literature, the complete reaction of PTFE with aluminum releases 8530 J/g of energy. Referring to Guo [37], the measured heat release was 6590 J/g, which is about 80% of the theoretical heat release. The theoretical heat release for the reaction of PTFE with magnesium is 9634 J/g, and the heat release from the decomposition of RDX is 1177 J/g. Table 5 shows the energy release data of the aluminum alloy reactive materials, indicating that the energy release gradually increases with the addition of metal powders (Mg/Zn). The energy release efficiency is highest when RDX is added to the reactive material, with an optimal energy release value of 47.03%. It shows that the energy release of the reactive material has a greater relationship with the material components.

The ∆*P* value reading in this paper is the highest peak point of the overpressure curve. Therefore, the peak point of the overpressure curve is used to calculate the energy release efficiency value, which may result in slightly higher efficiency calculation results. In addition, it can be seen that the accuracy of the test value of the total energy ∆E is very important for the calculation of the theoretical pressure $∆P^{*}$ on the basis of Formula (3).

### 3.5. The Impact Ignition Reaction Mechanism Analysis of PTFE-Based Reactive Materials

**Figure 12:**
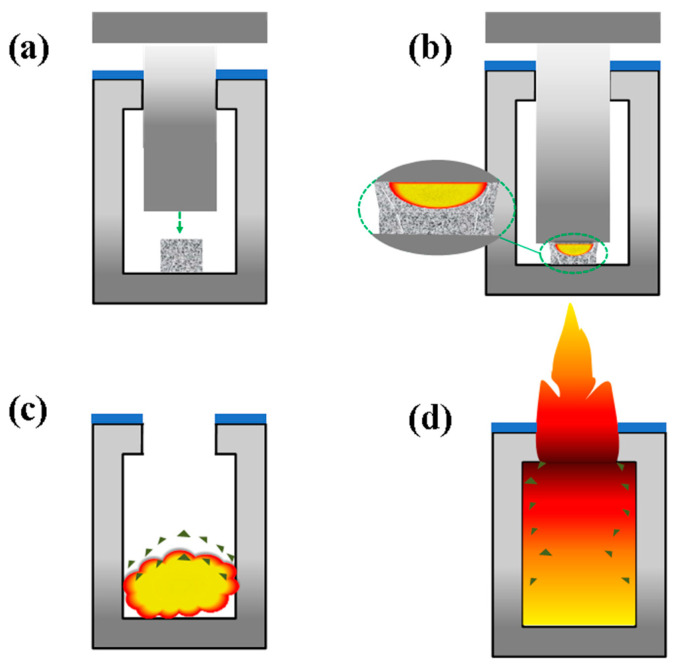
Schematic representation of the reaction mechanism of PTFE-based aluminum alloy reactive materials: (**a**) impact starts; (**b**) impact occurs; (**c**) impact ignition; (**d**) impact explosion.

The impact ignition reaction mechanism of the low-velocity impact response process of the aluminum alloy reactive material can be divided into the following four stages: (a) drop hammer impact; (b) impact-induced deflagration; (c) scattering of fragments and propagation of deflagration reaction; and (d) ejection of high-temperature gases and reaction product mixtures. The schematic diagram is shown in Figure 12, with a huge drop hammer impact, where aluminum alloy reactive material will undergo severe plastic deformation [21]. When the stress in the reactive material exceeds its compressive strength, the metal particles inside the aluminum alloy reactive material will undergo friction and slip, inducing tip cracks. The large deformation convergence of more heat makes the formation of energetic reaction materials accumulate easily and start the reaction point; that is, the “hotspot” [38] is generated. The closer to the hammer and anvil, the more likely the reactive material is to deform and produce more “hotspots”. Containing a large number of “hotspots” of reactive materials in the quasi-confined space of the deflagration reaction, the transparent quasi-closed container produces bright sparks. Due to the reaction-approximate adiabatic process, resulting in a rapid increase in the pressure inside the quasi-closed container, is the abovementioned “overpressure”.

The large-scale emergence of these hotspots is mainly attributed to the crucial role played by Zn particles in Al/Zn alloy powder and explosive crystal particles. The main ignition and energy release mechanism is the low-temperature vaporization of zinc or the high-temperature decomposition of explosives, which intensifies the fragmentation of Al_2_O_3_. A large number of hotspots are generated during the gas-phase combustion of Zn or the local detonation of explosives.

The low-melting-point Zn dispersed in the material rapidly vaporizes and absorbs heat under the adiabatic compression generated by impact. This process consumes energy and forms high-temperature and high-pressure regions. It also effectively destroys the oxide layer on the surface of Al particles, significantly lowering the initial ignition temperature of aluminum. As a result, the reaction rate and energy release efficiency are increased.

The RDX uniformly dispersed in the sample undergoes rapid thermal decomposition under the effects of shock waves and heat. The intense exothermic reaction produces numerous high-temperature regions, known as typical “hotspots.” These hotspots not only provide the necessary energy for the decomposition of PTFE, but the highly reactive free radicals generated by the impact process further promote the rapid propagation and sustained reaction of the entire system.

The formation of high-temperature and high-pressure areas caused by Zn, as well as hotspots produced by RDX, causes the accumulated heat to raise the aluminum particles to their reaction temperature. This facilitates a vigorous reaction between Al/Mg and PTFE. Therefore, PTFE-based reactive materials achieve higher impact energy release efficiency by modifying traditional base formulations.

### 3.6. Influencing Factor Analysis of Self-Designed Impact Energy Release Testing Device

In order to efficiently optimize the PTFE-based reactive materials, an independently designed impact energy release testing device was established. Many factors can affect the reliability of the experimental results of this device, mainly including the following points.

Impact energy determines whether the ignition threshold of the reaction can be exceeded. The low-velocity impact may only cause localized carbonization without triggering a chain reaction. High impact creates localized high-temperature and high-pressure zones, triggering global combustion. As impact energy increases, reaction delay time decreases, peak pressure and total released energy rise, and reaction completeness improves. However, excessively high impact energy may cause the reaction to occur too quickly, with more energy lost in the form of fragments.

High temperature generally lowers the ignition threshold and accelerates chemical kinetics, making exothermic reactions easier and more complete. Low temperature suppresses reaction rates and may require higher impact energy to ignite.

Humidity mainly affects reactions in two ways: firstly, by directly reacting with the metal powder surface to generate hydrogen or form an oxide layer, and secondly, by altering the powder’s surface adhesion and thermal conductivity. Overall, humidity usually reduces the activity of metal powders (especially aluminum), thereby raising the ignition threshold and decreasing the efficiency of energy release.

Chamber geometry determines gas expansion paths, as well as the reflection and dissipation of shock waves. Stronger confinement (small volume/rigid chamber) usually leads to higher peak pressure and more complete conversion of energy into mechanical work, whereas large volume or open systems cause energy to dissipate quickly, reduce peak pressure, and may result in extinguished or incomplete reactions.

## 4. Conclusions

PTFE-based reactive materials are taken as the research object and the influence of alloy powder and explosive additives on the energy release of reactive materials is explored. Based on the drop hammer impact test and the self-designed energy release testing device proposed by Ames, four PTFE-based different formulations of reactive materials are designed, especially the new formulation containing explosives. The energy release characteristics and ignition mechanism are analyzed. The following conclusions can be drawn.

1. Based on the impact response test by drop hammer, the ignition process of four PTFE-based reactive materials was captured using a high-speed camera. It was found that the samples 2#, 3#, and 4# had longer reaction durations compared to 1#. Samples 2#, 3# and 4# have a reaction duration of approximately 8 ms due to the addition of aluminum alloy powder or explosive additive. The four types of formulations’ ignition times are in the range of 0~0.9 ms. The degree of brightness of the combustion is as follows: 4# > 3# > 2# > 1#. Therefore, the addition of alloy powder and explosive is more advantageous to improving its energy release effect.
2. The changes in overpressure before and after the reaction of the reactive materials were quantitatively characterized in a quasi-confined space by means of the self-designed impact energy release testing device, namely a drop hammer impact response overpressure test system. The sorting relationship of the overpressure values of the four samples is summarized as follows: 4# > 3# > 2# > 1#, with overpressure values of 0.095, 0.063, 0.041, 0.031 MPa, respectively. The RDX-containing aluminum alloy reactive material 4# has the highest overpressure value. At the same time, its energy release efficiency is the highest among the four samples. Based on the combination of the characteristics of the drop height value, it was found that the addition of alloy metal powder will reduce the impact sensitivity, but when it is activated, it can effectively improve the destructive effect.
3. Based on the overpressure test results of the deflagration reaction of the PTFE-based aluminum alloy reactive materials, the energy release efficiency of formulation optimization is quickly obtained through the drop hammer impact energy release testing device without using conventional large-scale and high-speed ballistic impact tests. The impact ignition mechanism of PTFE-based reactive materials was proposed. This indicates that the small-scale drop hammer impact energy release testing system designed in this paper can provide a low-cost and efficient testing method and technical approach for the optimization and design research of reactive material formulations.

## Figures and Tables

**Table 1:** Sample number of PTFE aluminum alloy reactive material.

Serial Number	Component	Sample Percentage (%)
1#	PTFE/Al	73.5/26.5
2#	PTFE/(Al/Mg)	80/20
3#	PTFE/(Al/Mg/Zn)	80/20
4#	PTFE/(Al/Mg/Zn)/RDX	70/25/5

**Table 2:** Sensor parameters.

Parameter Name	Parameter Value
Company	Mingyu Electronics Co., Ltd., Mianyang, China
Type	MYD-1530 Piezoelectric Pressure Sensor
Range	0–125 kPa
Sensitivity	12.107 pC/kPa
Payload distance	244.7 pC
Uncertainties	1 kPa

**Table 3:** Initial time and duration of reactive ignition of reactive materials.

Serial Number	Initial Ignition Moment/ms	Reactive Duration/ms
1	0~0.9	5
2	0~0.9	8
3	0~0.9	8
4	0~0.9	9

**Table 4:** Overpressure test data table of four types of samples.

Num	Test Sample Number											
	1#/MPa			2#/MPa			3#/MPa			4#/MPa		
	$P_{1}$	$\mu_{1}$	$\sigma_{1}$	$P_{2}$	$\mu_{2}$	$\sigma_{2}$	$P_{3}$	$\mu_{3}$	$\sigma_{3}$	$P_{4}$	$\mu_{4}$	$\sigma_{4}$
1	0.0282	0.0306	0.0017	0.0394	0.0408	0.0009	0.0621	0.0631	0.0008	0.0928	0.0947	0.0015
2	0.0296			0.0407			0.0626			0.0934		
3	0.0310			0.0410			0.0630			0.0950		
4	0.0317			0.0413			0.0637			0.0957		
5	0.0323			0.0418			0.0642			0.0964		

Note: Overpressure test values, $P_{i}$; mean value, $\mu_{i}$; standard deviation, $\sigma_{i}$, i = 1, 2, 3, 4.

**Table 5:** Energy release data of aluminum alloy reactive material.

Serial Number	∆P (MPa)	${∆P}^{*}$ (MPa)	*η* (%)
1#	0.031	0.103	30.09
2#	0.041	0.163	25.15
3#	0.063	0.199	31.65
4#	0.095	0.202	47.03

## Data Availability

The data presented in this study are openly available.
